# Smoking and susceptibility to rheumatoid arthritis in a Swedish population-based case–control study

**DOI:** 10.1007/s10654-018-0360-5

**Published:** 2018-01-31

**Authors:** Anna Karin Hedström, Leszek Stawiarz, Lars Klareskog, Lars Alfredsson

**Affiliations:** 1 Institute of Environmental Medicine, Karolinska Institutet, Stockholm, Sweden; 2 Department of Clinical Neuroscience, Karolinska Institutet, Stockholm, Sweden; 3 Department of Medicine, Rheumatology Unit, Karolinska University Hospital, Solna, Stockholm, Sweden; 4 Centre for Occupational and Environmental Medine, Stockholm County Council, Stockholm, Sweden

**Keywords:** Rheumatoid arthritis, Smoking, Epidemiology, Anti-citrullinated peptide antibodies

## Abstract

Smoking is one of the most established risk factors for rheumatoid arthritis (RA). The aim of this study was to estimate how age at smoking debut, smoking cessation, duration, intensity, and cumulative dose of smoking influence the risk of developing anti-citrullinated peptide antibodies (ACPA) positive and ACPA negative RA. The present report is based on a Swedish population-based, case–control study with incident cases of RA (3655 cases, 5883 matched controls). Using logistic regression models, subjects with different smoking habits were compared regarding risk of developing the two variants of RA, by calculating odds ratios (OR) with 95% confidence intervals (CI). Smoking increased the risk of developing both ACPA positive (OR 1.9, 95% CI 1.7–2.1) and ACPA negative RA (OR 1.3, 95% CI 1.2–1.5). For both subsets of RA, there seemed to be a threshold (~ 2.5 pack years for ACPA positive RA and ~ 5 pack years for ACPA negative RA) below which no association between smoking and RA occurred. A dose–response association was observed between cumulative dose of smoking and risk of developing ACPA positive RA (*p* value for trend < 0.0001). Duration of smoking had a higher influence on the association between smoking and RA than did intensity of smoking. For both subsets of RA, the detrimental effect of smoking decreased after smoking cessation. Twenty years after smoking cessation, there was no longer an association between smoking and risk of ACPA negative RA, whereas the association between smoking and ACPA positive RA risk persisted and was dependent on the cumulative dose of smoking. Smoking increases the risk of both subsets of RA with a more pronounced influence on the risk of ACPA positive RA. Preventive measures in order to reduce smoking are essential and may result in a decline in RA incidence.

**Electronic supplementary material:**

The online version of this article (10.1007/s10654-018-0360-5) contains supplementary material, which is available to authorized users.

## Introduction

Rheumatoid arthritis (RA) is an immune-mediated inflammatory disease that occurs as a result of the interaction between genetic constitution and environmental triggers. The disease is subclassified into two major subsets based on presence of anti-citrullinated peptide antibodies (ACPA) [1–3]. The production of ACPA is mainly observed in patients carrying the shared epitope (SE) of HLA-DRB1. Both case–control and prospective cohort studies have demonstrated that smoking is the strongest environmental factor in RA development [3–6]. Smoking may induce citrullination of peptide antigens present in the lungs [7], and SE alleles interact with smoking in the triggering of anti-citrulline immunity that may lead to ACPA positive RA [8–11]. However, aspects of the association between smoking and RA, such as the effect of age at smoking debut, smoking cessation, duration, intensity and cumulative dose of smoking, require further elucidation.

Previous studies regarding the effect of smoking on ACPA negative RA have been inconclusive [12–14]. A number of studies have demonstrated that the risk of ACPA positive RA increases with increasing cumulative dose of smoking, but the shape of this dose–response association is unclear and it is unknown whether a certain degree of exposure is necessary for an association to occur [12–15]. It is also not established whether intensity or duration has the strongest effect on disease risk [13, 14, 16]. Smoking cessation has been observed to reduce the risk of RA associated with smoking, but reported results have been inconsistent regarding the persistence of the detrimental effect of smoking on RA risk after stopping smoking, especially in relation to the major subgroups of RA [13–15, 17]. To our knowledge, the effect of age at smoking debut has not previously been investigated. Further, some of the previous studies have been restricted to females, which makes studies of including both genders warranted.

In a Swedish population-based case–control study, we investigated the influence of smoking on the risk of developing ACPA positive RA and ACPA negative RA, and explored aspects of the association between smoking and RA risk that have previously been investigated only to a limited extent.

## Methods

### Study design and study subjects

This report was based on data from the ongoing project Epidemiological Investigation of Rheumatoid Arthritis (EIRA) which is a population-based case–control study comprising the population aged 18–70 years in the middle and southern parts of Sweden. A case was defined as a person in the study base with newly developed RA, defined according to the American College of Rheumatology criteria from 1987 [18]. All cases were evaluated by a rheumatologist. Twenty-one units, including all public hospital-based and almost all privately run rheumatology units in the study area participated in recruiting incident cases to the study. In connection to inclusion of a case, controls were randomly selected from the national population register, matched by age in 5-year age strata, gender and residential area (incidence density sampling). For cases recruited between November 1996 and October 2005, one control per case was selected (EIRA I). For cases recruited between October 2005 and September 2014, two controls per case were selected in order to increase power (EIRA II). If a control did not participate, a new control was selected using the same principles. During the study period November 1996–September 2014, completed questionnaires were obtained from 3724 cases and 5935 controls. The response proportion was 95% for the cases and 81% for the controls. The questionnaire used in EIRA II is very similar to than in EIRA I, but has more questions on some factors. For the present report, subjects who could not provide detailed information on smoking habits were excluded (26 cases and 52 controls). All aspects of the study were approved by the ethics committee of the Karolinska Institutet.

### APCA

Cases provided a blood sample at the clinic in which the case was entered. ACPA were measured as anti-CCP2 IgG using the commercial Immunoscan-RA MARK 2 ELISA test (Euro-Diagnostica AB, Malmö, Sweden), as described elsewhere [19]. All anti-CCP2 tests were carried out at Karolinska Institutet. According to the manufacturer´s instructions, an antibody level exceeding 25 AU/ml was regarded as ACPA positivity. ACPA status was missing for 43 cases and these were excluded.

### Data collection and definition of smoking

Information regarding life-style factors and different exposures was collected using a standardized questionnaire. Information on smoking was obtained by asking about current and previous smoking habits including duration of smoking, average number of cigarettes smoked per day, and type of cigarettes. For each case, the time of the initial appearance of RA symptoms was used as an estimate of the disease onset, and the year in which this occurred was defined as the index year. The corresponding controls were given the same index year. Information regarding smoking was considered prior to or during the index year in the cases and during the same period of time in the corresponding controls. Subjects who had smoked during the index year were defined as current smokers, those who had stopped smoking prior to the index year were defined as past smokers, and those who had never smoked before or during the index year were defined as never smokers. In order to analyze the influence of cumulative dose of smoking on the risk of developing the disease, we further categorized the smokers into groups based on the amount of cigarettes smoked (pack years) prior to index. One pack year is defined as having consumed 20 cigarettes per day for 1 year.

### Statistical analysis

Using logistic regression, the occurrence of ACPA positive- and ACPA negative RA in subjects with different smoking habits was compared with that in never smokers by calculating OR with 95% CI. The occurrence of RA among subjects who had started and stopped smoking in different life periods was compared with that among never smokers. Trend test for a dose response relationship regarding cumulative dose of smoking and risk of each subset of RA was performed by using a continuous variable for cumulative dose of smoking, expressed as pack-years, in a logistic regression model. In order to illustrate the influence of number of pack years on risk of each RA subset, we used polynomial regression of order 4 to fit the regression lines to the estimates of ORs. In order to determine whether intensity or duration of smoking contributes most to the risk of RA, we separately examined the components comprising pack years.

We performed matched analyses based on all available case–control pairs/triplets, as well as unmatched analyzes of the data based on all available cases and controls. Only the results from the unmatched analyses are presented in this report since these were in close agreement with those from the matched analyses but had a higher degree of precision in terms of more narrow confidence intervals.

All analyses were adjusted for age, residential area, study, and where appropriate gender. The OR’s were further adjusted for educational level (university degree or not), exposure to passive smoking (yes or no), alcohol consumption (number of drinks per week at study inclusion), body mass index at inclusion in the study (more or less than 25 kg/m^2^), and ancestry. Assessment of ancestry was based on whether the subject was born in Sweden or not, and whether either of the subject’s parents had immigrated to Sweden. A subject who was born in Sweden, whose parents had not immigrated, was classified as Swedish. All analyses were conducted using Statistical Analysis System (SAS) version 9.4.

## Results

Our analyses of smoking and RA risk included 3655 cases of RA (2339 ACPA positive cases and 1256 ACPA negative cases) and 5883 controls. Compared with never smokers, the OR for ACPA positive RA was 2.1 (95% CI 1.9–2.4) among current smokers, and 1.7 (95% CI 1.5–2.0) among past smokers (Table 1). The corresponding ORs for ACPA negative RA was 1.4 (95% CI 1.2–1.6) and 1.3 (95% CI 1.2–1.5). The increased risk of ACPA positive RA associated with smoking was more pronounced among men (OR 2.4, 95% CI 1.9–2.9) than among women (OR 1.8, 95% CI 1.6–2.0) whereas no significant gender differences were observed regarding ACPA negative RA. The corresponding results using conditional logistic regression are presented in Supplementary Table 1. The results are very similar to those in Table 1 based on unconditional logistic regression.

**Table 1:** Odds ratio (OR) with 95% confidence interval (95% CI) of developing ACPA positive and negative RA for different categories of smokers compared with never smokers

Total		ACPA positive RA				ACPA negative RA			
Total	ca/co^a^	OR (95% CI)^b^	OR (95% CI)^d^	ca/co^a^	OR (95% CI)^b^	OR (95% CI)^d^	ca/co^a^	OR (95% CI)^b^	OR (95% CI)^e^
Never	1210/2655	1.0 (reference)	1.0 (reference)	730/2655	1.0 (reference)	1.0 (reference)	480/2655	1.0 (reference)	1.0 (reference)
Past	1332/1909	1.7 (1.5–1.8)	1.7 (1.5–1.8)	884/1909	1.7 (1.5–1.9)	1.7 (1.5–2.0)	448/1909	1.3 (1.1–1.5)	1.3 (1.1–1.5)
Current	1113/1319	1.9 (1.7–2.1)	1.8 (1.6–2.0)	785/1319	2.2 (1.9–2.5)	2.1 (1.9–2.4)	328/1319	1.4 (1.2–1.6)	1.4 (1.2–1.6)
Ever	2445/3228	1.5 (1.4–1.7)	1.5 (1.4–1.7)	1669/3228	1.9 (1.7–2.1)	1.9 (1.7–2.1)	776/3228	1.3 (1.2–1.5)	1.3 (1.2–1.5)
Women	ca/co^a^	OR (95% CI)^c^	OR (95% CI)^e^	ca/co^a^	OR (95% CI)^c^	OR (95% CI)^e^	ca/co^a^	OR (95% CI)^c^	OR (95% CI)^e^
Never	923/1956	1.0 (reference)	1.0 (reference)	574/1956	1.0 (reference)	1.0 (reference)	349/1956	1.0 (reference)	1.0 (reference)
Past	903/1290	1.5 (1.3–1.7)	1.5 (1.3–1.7)	606/1290	1.6 (1.4–1.8)	1.6 (1.4–1.9)	297/1290	1.3 (1.1–1.5)	1.3 (1.1–1.5)
Current	799/973	1.7 (1.5–2.0)	1.7 (1.5–1.9)	565/973	2.0 (1.7–2.3)	1.9 (1.7–2.2)	234/973	1.4 (1.1–1.6)	1.4 (1.1–1.6)
Ever	1702/2263	1.6 (1.4–1.8)	1.6 (1.4–1.8)	1171/2263	1.8 (1.6–2.0)	1.8 (1.6–2.0)	531/2263	1.3 (1.1–1.5)	1.3 (1.1–1.5)
Men	ca/co^a^	OR (95% CI)^c^	OR (95% CI)^e^	ca/co^a^	OR (95% CI)^c^	OR (95% CI)^e^	ca/co^a^	OR (95% CI)^c^	OR (95% CI)^e^
Never	287/699	1.0 (reference)	1.0 (reference)	156/699	1.0 (reference)	1.0 (reference)	131/699	1.0 (reference)	1.0 (reference)
Past	429/619	1.7 (1.4–2.1)	1.7 (1.4–2.1)	278/619	2.2 (1.7–2.7)	2.1 (1.7–2.7)	151/619	1.2 (0.9–1.6)	1.2 (0.9–1.5)
Current	314/346	2.2 (1.8–2.7)	2.1 (1.7–2.6)	220/346	2.9 (2.3–2.7)	2.8 (2.2–3.5)	94/346	1.5 (1.1–2.0)	1.5 (1.0–1.9)
Ever	743/965	1.9 (1.6–2.3)	1.8 (1.5–2.2)	498/965	2.5 (2.0–3.0)	2.4 (1.9–2.9)	245/965	1.3 (1.0–1.7)	1.3 (1.0–1.7)

^a^Number of cases and controls

^b^Adjusted for age, gender, residential area, and study

^c^Adjusted for age, residential area, and study

^d^Adjusted for age, gender, residential area, study, ancestry, educational level, passive smoking, alcohol consumption, and body mass index at inclusion in the study

^e^Adjusted for age, residential area, study, ancestry, educational level, passive smoking, alcohol consumption, and body mass index at inclusion in the study

**Fig. 1:**
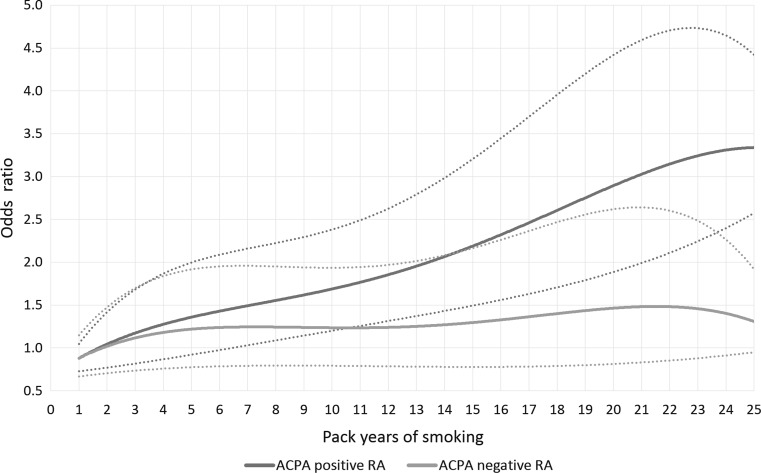
Dose-response relationship between cumulative dose of smoking and risk of ACPA positive RA and ACPA negative RA

Both with regard to risk of ACPA positive and ACPA negative RA, there seemed to be a threshold (~ 2.5 pack years for ACPA positive RA and ~ 5 pack years for ACPA negative RA) below which no association between smoking and RA occurred (Table 2). A dose–response association was observed between cumulative dose of smoking (exceeding five pack years) and risk of developing ACPA positive RA (*p* value for trend < 0.0001). In Fig. 1, we illustrate the influence of number of pack years on risk of each RA subset.

**Table 2:** OR with 95% CI of developing ACPA positive RA and ACPA negative RA for ever smokers compared with never smokers, by cumulative dose of smoking

Pack years	ACPA positive RA				ACPA negative RA			
	ca/co^a^	OR (95% CI)^b^	OR (95% CI)^c^	*p*	ca/co^a^	OR (95% CI)^b^	OR (95% CI)^c^	*p*
0	730/2655	1.0 (reference)	1.0 (reference)		480/2655	1.0 (reference)	1.0 (reference)	
1–2.5	160/551	1.0 (0.9–1.2)	1.0 (0.8–1.2)	0.9	101/551	1.0 (0.8–1.3)	1.0 (0.8–1.3)	0.8
2.5–5	127/362	1.2 (1.0–1.5)	1.2 (1.0–1.6)	0.05	66/362	1.0 (0.7–1.3)	1.0 (0.8–1.3)	0.9
5–7.5	102/261	1.4 (1.1–1.8)	1.4 (1.1–1.8)	0.007	70/261	1.4 (1.1–1.9)	1.4 (1.1–2.0)	0.007
7.5–10	117/257	1.7 (1.3–2.1)	1.6 (1.3–2.0)	< 0.0001	62/257	1.3 (1.0–1.7)	1.3 (1.0–1.7)	0.1
10–12.5	97/215	1.7 (1.3–2.2)	1.7 (1.3–2.2)	< 0.0001	41/215	1.0 (0.7–1.4)	1.0 (0.7–1.4)	0.8
12.5–15	126/214	2.2 (1.8–2.8)	2.2 (1.7–2.8)	< 0.0001	46//214	1.1 (0.8–1.6)	1.1 (0.8–1.6)	0.4
15–17.5	98/159	2.4 (1.8–3.1)	2.3 (1.8–3.1)	< 0.0001	48/159	1.6 (1.1–2.2)	1.6 (1.1–2.2)	0.009
17.5–20	122/182	2.6 (2.1–3.4)	2.6 (2.0–3.3)	< 0.0001	51/182	1.5 (1.1–2.0)	1.5 (1.1–2.0)	0.03
> 20	652/870	3.0 (2.7–3.5)	3.0 (2.6–3.4)	< 0.0001	244/870	1.5 (1.2–1.7)	1.5 (1.2–1.7)	0.0002
			Trend < 0.0001				Trend < 0.0001	

^a^Number of cases and controls

^b^Adjusted for age, gender, residential area, and study

^c^Adjusted for age, gender, residential area, study, ancestry, educational level, passive smoking, alcohol consumption, and body mass index at inclusion in the study

Duration of smoking had a stronger influence on the association between smoking and RA than did intensity of smoking (Table 3) and there was no significant association between smoking and RA risk among those who had smoked less than 10 years, regardless of the intensity of smoking. Subjects who had smoked longer than 20 years had an almost threefold increased risk of ACPA positive RA, and a 60% increased risk of ACPA negative RA, regardless of the intensity of smoking. In Supplementary Tables 2 and 3, we present the results when intensity and duration are considered separately.

**Table 3:** OR with 95% CI of developing ACPA positive RA and ACPA negative RA for smokers compared with never-smokers, by duration (years) and intensity of smoking (number of cigarettes smoked daily)

ACPA positive RA								
Intensity Duration		0		< 10		10–20		> 20
0	730/2655	1.0 (reference)						
0–10			77/268	1.0 (0.8–1.2)	78/262	1.4 (1.2–1.8)	264/257	1.6 (1.3–2.1)
11–19			68/222	1.2 (0.9–1.7)	126/311	1.6 (1.2–2.2)	555/777	1.7 (1.2–2.4)
20–			34/109	2.8 (2.3–3.4)	74/188	2.9 (2.5–3.4)	325/476	2.8 (2.3–3.3)

ACPA negative RA								
Intensity Duration		0		< 10		10–20		> 20
0	480/2655	1.0 (reference)						
0–10			52/268	1.1 (0.8–1.5)	57/262	1.0 (0.7–1.2)	128/457	0.9 (0.6–1.2)
11–19			34/222	1.3 (0.9–1.9)	76/311	1.3 (0.9–1.9)	200/777	1.3 (0.8–2.0)
20–			12/109	1.6 (1.3–2.1)	128/188	1.6 (1.3–2.0)	134/476	1.6 (1.2–2.0)

Adjusted for age, gender, residential area, study, ancestry, educational level, passive smoking, alcohol consumption, and body mass index at inclusion in the study

Age at smoking debut had no influence on the association between smoking and RA risk when pack years of smoking was taken into consideration (Table 4) and no specific time window of smoking seemed more critical than the other (data not shown). We present the effect of age at smoking debut without considering number of pack years in Supplementary Table 4.

**Table 4:** OR with 95% CI of developing ACPA positive RA and ACPA negative RA for smokers compared with never smokers, by age at smoking debut and cumulative dose of smoking

ACPA positive RA								
Pack years of smoking Age at smoking debut	0			< 10		10–20		> 20
0	730/2655	1.0 (reference)						
< 20			242/689	1.2 (1.0–1.4)	302/530	2.1 (1.8–2.5)	497/637	3.1 (2.7–3.4)
20–25			115/310	1.3 (1.1–1.7)	104/212	2.0 (1.6–2.6)	133/183	3.0 (2.4–3.9)
> 25			129/382	1.3 (1.0–1.6)	56/78	3.0 (2.0–4.3)	23/50	1.9 (1.1–3.2)

ACPA negative RA								
Pack years of smoking Age at smoking debut	0			< 10		10–20		> 20
0	480/2655	1.0 (reference)						
< 20			150/689	1.2 (1.0–1.5)	119/530	1.2 (1.0–1.5)	181/637	1.5 (1.2–1.8)
20–25			72/310	1.3 (1.0–1.8)	57/212	1.4 (1.1–2.0)	49/183	1.4 (1.0–1.9)
> 25			69/382	1.1 (0.9–1.4)	18/78	1.2 (0.7–2.0)	14/50	1.5 (0.8–2.7)

Adjusted for age, gender, residential area, study, ancestry, educational level, passive smoking, alcohol consumption, and body mass index at inclusion in the study

Among both subsets of RA, the detrimental effect of smoking decreased after smoking cessation. Twenty years after smoking cessation, there was no longer an association between smoking and ACPA negative RA risk, whereas the association between smoking and ACPA positive RA risk persisted and was dependent on the cumulative dose of smoking (Table 5). Supplementary Table 5 shows the association between smoking and RA after smoking cessation without taking the cumulative dose of smoking into consideration. In all analyses, the risk of RA associated with smoking was generally higher among men than among women.

**Table 5:** OR with 95% CI of developing ACPA positive and negative RA for smokers compared with never-smokers, by number of years since stopping smoking and cumulative dose of smoking

ACPA positive RA								
Pack years of smoking Years since stopping smoking	0			< 10		10–20		> 20
Never smokers	730/2655	1.0 (reference)						
Current smokers			146/411	1.2 (1.0–1.4)	227/325	2.5 (2.1–3.1)	382/518	2.8 (2.4–3.3)
< 10 years			114/254	1.5 (1.2–1.9)	109/149	2.7 (2.1–3.6)	185/181	4.0 (3.2–5.1)
10–20 years			92/250	1.4 (1.1–1.8)	81/188	1.7 (1.3–2.6)	63/114	2.3 (1.7–3.2)
> 20 years			133/466	1.2 (0.9–1.4)	46/158	1.2 (0.9–1.8)	23/57	1.7 (1.0–2.8)

ACPA negative RA								
Pack years of smoking Years since stopping smoking	0			< 10		10–20		> 20
Never smokers	480/2655	1.0 (reference)						
Current smokers			81/411	1.2 (0.9–1.6)	89/325	1.5 (1.2–1.9)	143/518	1.5 (1.1–1.8)
< 10 years			50/254	1.2 (0.9–1.7)	46/149	1.7 (1.1–2.3)	57/181	1.6 (1.2–2.3)
10–20 years			56/250	1.3 (1.0–1.8)	37/188	1.1 (0.7–1.6)	34/114	1.5 (1.0–2.3)
> 20 years			104/466	1.2 (0.9–1.5)	22/158	0.8 (0.5–1.2)	10/57	0.9 (0.5–1.7)

Adjusted for age, gender, residential area, study, ancestry, educational level, passive smoking, alcohol consumption, and body mass index at inclusion in the study

## Discussion

In the present report we aimed to extend previous knowledge by analyzing aspects of the association between smoking and RA that have been less studied. Smoking is the most established environmental risk factor for ACPA positive RA risk, whereas previous studies regarding the effect of smoking on ACPA negative RA have been inconclusive [12–14]. In our present report, we observed that smoking was associated with increased risk of developing both subsets of RA.

Most previous studies investigating the dose response relationship between smoking and RA risk have suggested that the impact of smoking on RA is limited to those with a cumulative exposure exceeding 10 pack years [12–14], whereas other studies have suggested an increased risk of RA even among light smokers with a cumulative dose of smoking less than 10 pack years [15]. Both with regard to ACPA positive RA and ACPA negative RA risk, our results indicate the existence of a threshold below which no association between smoking and RA occur. Further we were able to describe the dose response relationship in more detail compared to previous studies.

Our results regarding duration and intensity of smoking on the risk of RA are in accordance with previous studies, which found that duration of smoking has a stronger impact on RA development than intensity of smoking [13, 14, 16]. We found no significant association between smoking and RA risk among those who had smoked less than 10 years, regardless of the intensity of smoking. Subjects who had smoked longer than 20 years had an almost threefold increased risk of ACPA positive RA, and a 60% increased risk of ACPA negative RA, regardless of the intensity of smoking.

Smoking cessation has been observed to reduce the risk of RA associated with smoking, but reported results have been inconsistent regarding the persistence of the detrimental effect of smoking on RA risk after stopping smoking [13–15, 17]. None of the previous studies had the opportunity to investigate the influence of smoking cessation on the risk of ACPA positive RA and ACPA negative RA separately. Among both subsets of RA, we found that the influence of smoking on RA risk decreased after smoking cessation. Twenty years after smoking cessation, there was no longer an association between smoking and ACPA negative RA risk, whereas the association between smoking and ACPA positive RA risk persisted and was dependent on the cumulative dose of smoking.

The effect of age at smoking debut has not previously been investigated. Age at smoking debut had no influence on the association between smoking and RA risk when pack years of smoking was taken into consideration and no specific time window of smoking seemed more critical than the other.

The exact mechanisms responsible for the association between smoking and ACPA negative RA are not yet fully understood. One explanation to this association may be the fact that some of the anti-CCP2 negative individuals indeed have reactivity against citrullinated proteins/peptides not captured by the CCP2 assay [20, 21] and thus that similar mechanisms as in anti-CCP2 positive individuals may be active in a subset of anti-CCP2 negative patients. Other explanations must of course also be considered and it is for example known that both humoral and cell-mediated immunity are affected by smoking in a more general sense [22], and that smokers have increased levels of important mediators of inflammation such as Interleukin-6 [23]. Smoking also increases the oxidative stress and activates endogenous sources of free radicals which may contribute to RA etiology [24, 25].

Smoking has been estimated to be responsible for 20% of RA cases overall and with one-third of cases of ACPA positive RA [26]. Smoking is also associated with other negative health effects such as cancers, cardiovascular and respiratory disease, and preventive measures in order to reduce smoking are essential [27]. Prevention programs aiming at smoking cessation and the reform of tobacco legislation in Finland have resulted in long-term prevention of cardiovascular disease [28] and by a similar decline in RA incidence [29].

Our study was designed as a case–control study with incident cases, and information regarding smoking habits and exposure to passive smoking was collected retrospectively. Even though recall bias could still be of concern, we tried to minimize this risk by using incident cases of RA. We took great effort to obtain information on lifestyle factors and environmental exposures in an identical way for the cases and the controls. Furthermore, the questionnaire contained a wide range of questions regarding many potential environmental risk factors and no section in the questionnaire was given prime focus.

With regard to the degree of a differential recall, it is worth considering the different dose response associations between smoking and ACPA positive RA and ACPA negative RA, respectively. On the assumption that the quality of reporting smoking habits is the same for ACPA positive- and ACPA negative cases (which is highly likely), and further, that the true association between smoking and ACPA negative RA is null (i.e. the observed association between smoking and ACPA negative RA is totally due to recall bias), then we can get an estimation of the maximal degree of the recall bias. Based on this we still can observe a strong dose–response relationship between smoking and ACPA positive RA. However, we think it is highly unlikely that the observed increased risk for ACPA negative RA among ever smokers, [OR 1.3 (95% CI 1.2–1.5)] is totally due do reporting error with regard to having ever smoked.

A potential selection bias may arise when recruiting cases and controls. The proportion of respondents with regard to participation in EIRA was 95% for cases and 81% for controls. Since the structure of the Swedish public health care system provides equal access to medical services for all Swedish citizens, it is most likely that almost all cases of RA are referred to public rheumatology units and it is not likely that the few unidentified cases would cause a substantial bias in our calculations. Selection bias among controls is likely to be modest since the prevalence of smoking among controls, seen as an indicator of life style, was in line with that of the general population at equivalent ages [30].

In summary, in this population-based case–control study of RA, we demonstrate that smoking is associated with increased risk of both subsets of RA. There seems to be a threshold below which no association between smoking and RA occurs. After smoking cessation, the detrimental effect of smoking has a more persistent influence on the risk of ACPA positive RA.

## Electronic supplementary material

Below is the link to the electronic supplementary material.

Supplementary material 1 (DOC 43 kb)
