# Supplementary material for: Smoking and susceptibility to rheumatoid arthritis in a Swedish population-based case–control study
Source: Eur J Epidemiol. 2018 Jan 31;33(4):415–23. doi: 10.1007/s10654-018-0360-5 (PMC5945793; doi:10.1007/s10654-018-0360-5)
Supplement: Supplementary file 1 — Supplementary material 1 (DOC 43 kb) [file 10654_2018_360_MOESM1_ESM.doc]

**Supplementary table 1.** Odds ratio (OR) with 95% confidence interval (95% CI) of developing ACPA positive and negative RA for different categories of smokers compared with never smokers. Conditional logistic regression.
 **Total ACPA positive RA ACPA negative RA**

**Total ca/co* OR (95% CI)# p ca/co* OR (95% CI)# p ca/co* OR (95% CI)# p**

Never 1158/2290 1.0 (reference) 701/2290 1.0 (reference) 457/2290 1.0 (reference)

Past 1217/1564 1.6 (1.4-1.9) <0.0001 816/1564 1.8 (1.6-2.0) <0.0001 401/1564 1.2 (1.1-1.5) 0.03

Current 1035/1073 1.9 (1.7-2.3) <0.0001 728/1073 2.0 (1.8-2.3) <0.0001 307/1073 1.4 (1.1-1.6) 0.005

Ever 2252/2637 1.6 (1.4-1.9) <0.0001 1544/2637 1.8 (1.5-2.0) <0.0001 708/2637 1.3 (1.1-1.5) 0.003

**Women** **ca/co* OR (95% CI)**¤ **p ca/co* OR (95% CI)**¤ **p** **ca/co* OR (95% CI)**¤ **p**

Never 886/1697 1.0 (reference) 551/1697 1.0 (reference) 335/1697 1.0 (reference)

Past 812/1049 1.5 (1.3-1.7) <0.0001 550/1049 1.5 (1.4-1.9) <0.0001 262/1049 1.3 (1.1-1.5) 0.02

Current 745/795 1.7 (1.5-1.9) <0.0001 525/795 1.8 (1.6-2.1) <0.0001 220/795 1.4 (1.1-1.6) 0.008

Ever 1557/1844 1.6 (1.3-1.7) <0.0001 1075/1844 1.7 (1.5-2.0) <0.0001 482/1844 1.3 (1.0-1.5) 0.003

**Men** **ca/co* OR (95% CI)**¤ **p ca/co* OR (95% CI)**¤ **p ca/co* OR (95% CI)**¤ **p**

Never 272/593 1.0 (reference) 150/593 1.0 (reference) 122/593 1.0 (reference)

Past 405/515 1.7 (1.4-1.9) <0.0001 266/515 2.1 (1.6-2.8) <0.0001 139/515 1.1 (0.8-1.5) 0.9

Current 290/278 2.0 (1.6-2.5) <0.0001 203/278 2.8 (2.1-3.7) <0.0001 87/278 1.2 (0.9-1.8) 0.3

Ever 695/793 1.8 (1.4-2.2) <0.0001 469/793 2.3 (1.8-2.9) <0.0001 226/793 1.2 (0.8-1.6) 0.5

* number of cases and controls
# adjusted for age, gender, residential area, ancestry, educational level, passive smoking, alcohol consumption, and body mass index at inclusion in the study.
¤ adjusted for age, residential area, ancestry, educational level, passive smoking, alcohol consumption, and body mass index at inclusion in the study.

**Supplementary table 2.** OR with 95% CI of developing ACPA positive RA and ACPA negative RA for ever smokers compared with never smokers, by intensity of smoking.

**ACPA positive RA ACPA negative RA**

**Intensity** **ca/co* OR (95% CI)# p ca/co* OR (95% CI)# p**

Never smoking 730/2655 1.0 (reference) 480/2655 1.0 (reference)

0-10 419/988 1.6 (1.4-1.8) <0.0001 237/988 1.4 (1.1-1.6) 0.0006

11-19 749/1310 2.1 (1.8-2.4) <0.0001 310/1310 1.3 (1.1-1.5) 0.004

20- 433/773 2.1 (1.8-2.4) <0.0001 182/773 1.3 (1.0-1.5) 0.02

* number of cases and controls
# adjusted for age, gender, residential area, ancestry, educational level, passive smoking, alcohol consumption, and body mass index at inclusion in the study.

**Supplementary table 3.** OR with 95% CI of developing ACPA positive RA and ACPA negative RA for ever smokers compared with never smokers, by duration of smoking.

**ACPA positive RA ACPA negative RA**

**Duration** **ca/co* OR (95% CI)# p ca/co* OR (95% CI)# p**

Never smoking 730/2655 1.0 (reference) 730/2655 1.0 (reference)

0-10 505/1393 1.1 (0.9-1.3) 0.9 249/1393 1.0 (0.8-1.2) 0.9

11-19 194/455 1.4 (1.1-1.7) 0.001 107/455 1.2 (1.0-1.5) 0.003

20- 902/1223 2.8 (2.4-3.3) <0.0001 373/1223 1.4 (1.2-1.7) <0.0001

* number of cases and controls
# adjusted for age, gender, residential area, ancestry, educational level, passive smoking, alcohol consumption, and body mass index at inclusion in the study.

**Supplementary table 4.** OR with 95% CI of developing ACPA positive RA and ACPA negative RA for ever smokers compared with never smokers, by age at smoking debut.

**ACPA positive RA ACPA negative RA**

**Age at smoking debut** **ca/co* OR (95% CI)# p ca/co* OR (95% CI)# p**

Never smoking 730/2655 1.0 (reference) 480/2655 1.0 (reference)

0-19 486/1381 2.0 (1.8-2.3) <0.0001 291/1381 1.3 (1.1-1.5) 0.0002

20-25 463/820 1.9 (1.6-2.2) <0.0001 194/820 1.4 (1.1-1.7) 0.002

26- 652/870 1.5 (1.3-1.8) <0.0001 244/870 1.1 (0.9-1.4) 0.4

* number of cases and controls
# adjusted for age, gender, residential area, ancestry, educational level, passive smoking, alcohol consumption, and body mass index at inclusion in the study.

**Supplementary table 5.** OR with 95% CI of developing ACPA positive RA and ACPA negative RA for ever smokers compared with never smokers, by years since stopping smoking.

**ACPA positive RA ACPA negative RA**

**Years since stopping smoking** **ca/co* OR (95% CI)# p ca/co* OR (95% CI)# p**

Never smoking 730/2655 1.0 (reference) 480/2655 1.0 (reference)

Current 755/1254 2.1 (1.9-2.4) 313/1254 1.4 (1.2-1.6) 0.0001

0-10 408/584 2.5 (2.2-2.9) 153/584 1.4 (1.2-1.8) 0.0005

11-19 236/552 1.6 (1.4-1.9) 127/552 1.3 (1.0-1.6) 0.03

20- 202/681 1.1 (0.9-1.3) 136/681 1.0 (0.8-1.3) 0.7

* number of cases and controls
# adjusted for age, gender, residential area, ancestry, educational level, passive smoking, alcohol consumption, and body mass index at inclusion in the study.
